# Supplementary material for: Modeling glioblastoma heterogeneity as a dynamic network of cell states
Source: Mol Syst Biol. 2021 Sep 16;17(9):e10105. doi: 10.15252/msb.202010105 (PMC8444284; doi:10.15252/msb.202010105)
Supplement: Supplementary file 6 — Source Data for Figure 5 [file MSB-17-e10105-s004.zip › Figure5A_sourcedata/GSEA_3071/nosigdb_stateIV.GseaPreranked.1622795164626/RICHARDS_NOWAKOWSKI_SCIENCE_2017_ASTROCYTE_UPREG.html]

Details for gene set RICHARDS\_NOWAKOWSKI\_SCIENCE\_2017\_ASTROCYTE\_UPREG[GSEA]

|  || Dataset | state53071 |
| Phenotype | NoPhenotypeAvailable |
| Upregulated in class | na\_pos |
| GeneSet | RICHARDS\_NOWAKOWSKI\_SCIENCE\_2017\_ASTROCYTE\_UPREG |
| Enrichment Score (ES) | 0.44979408 |
| Normalized Enrichment Score (NES) | 3.0077264 |
| Nominal p-value | 0.0 |
| FDR q-value | 0.0 |
| FWER p-Value | 0.0 |
Table: GSEA Results Summary

  

Fig 1: Enrichment plot: RICHARDS\_NOWAKOWSKI\_SCIENCE\_2017\_ASTROCYTE\_UPREG      
 Profile of the Running ES Score & Positions of GeneSet Members on the Rank Ordered List

  

| PROBE | GENE SYMBOL | GENE\_TITLE | RANK IN GENE LIST | RANK METRIC SCORE | RUNNING ES | CORE ENRICHMENT || 1 | PDGFRA |  |  | 1 | 2.924 | 0.0096 | Yes |
| 2 | C1orf61 |  |  | 3 | 2.760 | 0.0187 | Yes |
| 3 | GFAP |  |  | 6 | 2.698 | 0.0274 | Yes |
| 4 | ID4 |  |  | 7 | 2.594 | 0.0361 | Yes |
| 5 | PTPRZ1 |  |  | 10 | 2.539 | 0.0443 | Yes |
| 6 | PMP2 |  |  | 11 | 2.494 | 0.0526 | Yes |
| 7 | S100B |  |  | 12 | 2.461 | 0.0609 | Yes |
| 8 | FABP7 |  |  | 14 | 2.398 | 0.0688 | Yes |
| 9 | IGFBP2 |  |  | 16 | 2.364 | 0.0765 | Yes |
| 10 | GPM6B |  |  | 21 | 2.210 | 0.0832 | Yes |
| 11 | PTN |  |  | 25 | 2.140 | 0.0897 | Yes |
| 12 | FAM181B |  |  | 26 | 2.105 | 0.0968 | Yes |
| 13 | CLU |  |  | 27 | 2.064 | 0.1038 | Yes |
| 14 | SCRG1 |  |  | 28 | 2.056 | 0.1107 | Yes |
| 15 | LRRN3 |  |  | 29 | 2.015 | 0.1175 | Yes |
| 16 | CXCL14 |  |  | 34 | 2.003 | 0.1234 | Yes |
| 17 | MAP3K1 |  |  | 42 | 1.903 | 0.1284 | Yes |
| 18 | CADM4 |  |  | 47 | 1.860 | 0.1338 | Yes |
| 19 | SLC1A2 |  |  | 48 | 1.860 | 0.1401 | Yes |
| 20 | LIMA1 |  |  | 58 | 1.805 | 0.1443 | Yes |
| 21 | TNC |  |  | 66 | 1.751 | 0.1488 | Yes |
| 22 | SYT11 |  |  | 67 | 1.740 | 0.1547 | Yes |
| 23 | DCLK2 |  |  | 74 | 1.704 | 0.1592 | Yes |
| 24 | CLSTN1 |  |  | 77 | 1.650 | 0.1643 | Yes |
| 25 | SLC35F1 |  |  | 78 | 1.647 | 0.1699 | Yes |
| 26 | ZBTB20 |  |  | 81 | 1.628 | 0.1749 | Yes |
| 27 | CHPT1 |  |  | 87 | 1.614 | 0.1793 | Yes |
| 28 | ITPR2 |  |  | 88 | 1.610 | 0.1848 | Yes |
| 29 | MMP16 |  |  | 90 | 1.578 | 0.1899 | Yes |
| 30 | NPNT |  |  | 91 | 1.569 | 0.1952 | Yes |
| 31 | FJX1 |  |  | 92 | 1.565 | 0.2004 | Yes |
| 32 | GPM6A |  |  | 98 | 1.538 | 0.2046 | Yes |
| 33 | TGFB2 |  |  | 108 | 1.511 | 0.2078 | Yes |
| 34 | MYO10 |  |  | 121 | 1.484 | 0.2104 | Yes |
| 35 | LRRN1 |  |  | 122 | 1.482 | 0.2154 | Yes |
| 36 | PTPRG |  |  | 123 | 1.479 | 0.2204 | Yes |
| 37 | TRIM9 |  |  | 125 | 1.470 | 0.2251 | Yes |
| 38 | FGFBP3 |  |  | 127 | 1.465 | 0.2298 | Yes |
| 39 | LSAMP |  |  | 130 | 1.454 | 0.2343 | Yes |
| 40 | RNF180 |  |  | 132 | 1.446 | 0.2390 | Yes |
| 41 | RASSF2 |  |  | 140 | 1.400 | 0.2423 | Yes |
| 42 | GPRC5B |  |  | 142 | 1.390 | 0.2467 | Yes |
| 43 | ID2 |  |  | 149 | 1.378 | 0.2502 | Yes |
| 44 | SEL1L3 |  |  | 151 | 1.371 | 0.2546 | Yes |
| 45 | BCHE |  |  | 155 | 1.362 | 0.2586 | Yes |
| 46 | DUSP6 |  |  | 156 | 1.359 | 0.2631 | Yes |
| 47 | GNG7 |  |  | 157 | 1.350 | 0.2677 | Yes |
| 48 | RHOBTB3 |  |  | 169 | 1.322 | 0.2699 | Yes |
| 49 | ETV5 |  |  | 174 | 1.305 | 0.2735 | Yes |
| 50 | NCAN |  |  | 181 | 1.298 | 0.2766 | Yes |
| 51 | CDCA7L |  |  | 185 | 1.292 | 0.2804 | Yes |
| 52 | SOX8 |  |  | 199 | 1.263 | 0.2820 | Yes |
| 53 | SOX2 |  |  | 212 | 1.231 | 0.2837 | Yes |
| 54 | SOX9 |  |  | 214 | 1.223 | 0.2876 | Yes |
| 55 | LDLRAD3 |  |  | 216 | 1.218 | 0.2915 | Yes |
| 56 | FABP5 |  |  | 230 | 1.187 | 0.2928 | Yes |
| 57 | ITM2B |  |  | 233 | 1.181 | 0.2964 | Yes |
| 58 | NOVA1 |  |  | 234 | 1.181 | 0.3004 | Yes |
| 59 | PRCP |  |  | 242 | 1.169 | 0.3029 | Yes |
| 60 | SEMA5A |  |  | 247 | 1.153 | 0.3060 | Yes |
| 61 | LITAF |  |  | 252 | 1.144 | 0.3090 | Yes |
| 62 | WSCD1 |  |  | 253 | 1.141 | 0.3128 | Yes |
| 63 | PCDHGC3 |  |  | 255 | 1.133 | 0.3165 | Yes |
| 64 | SLC1A3 |  |  | 268 | 1.107 | 0.3177 | Yes |
| 65 | MEIS1 |  |  | 269 | 1.107 | 0.3215 | Yes |
| 66 | NLGN1 |  |  | 282 | 1.069 | 0.3226 | Yes |
| 67 | NLGN4X |  |  | 284 | 1.068 | 0.3260 | Yes |
| 68 | STON2 |  |  | 285 | 1.064 | 0.3296 | Yes |
| 69 | CD9 |  |  | 288 | 1.062 | 0.3328 | Yes |
| 70 | SRI |  |  | 298 | 1.037 | 0.3344 | Yes |
| 71 | PCDH9 |  |  | 301 | 1.034 | 0.3375 | Yes |
| 72 | SALL3 |  |  | 306 | 1.029 | 0.3402 | Yes |
| 73 | ITGB8 |  |  | 307 | 1.027 | 0.3436 | Yes |
| 74 | FAT1 |  |  | 311 | 1.023 | 0.3465 | Yes |
| 75 | CACNB2 |  |  | 327 | 0.992 | 0.3467 | Yes |
| 76 | ST3GAL5 |  |  | 336 | 0.985 | 0.3484 | Yes |
| 77 | STK17A |  |  | 337 | 0.982 | 0.3517 | Yes |
| 78 | LYPD1 |  |  | 338 | 0.980 | 0.3550 | Yes |
| 79 | DBI |  |  | 339 | 0.980 | 0.3583 | Yes |
| 80 | MAGI1 |  |  | 355 | 0.969 | 0.3586 | Yes |
| 81 | SPECC1 |  |  | 360 | 0.960 | 0.3610 | Yes |
| 82 | MLC1 |  |  | 366 | 0.946 | 0.3631 | Yes |
| 83 | BAALC |  |  | 367 | 0.944 | 0.3663 | Yes |
| 84 | SESN3 |  |  | 380 | 0.925 | 0.3670 | Yes |
| 85 | ZEB1 |  |  | 385 | 0.920 | 0.3693 | Yes |
| 86 | OLIG1 |  |  | 391 | 0.914 | 0.3713 | Yes |
| 87 | TRIB2 |  |  | 396 | 0.911 | 0.3736 | Yes |
| 88 | P2RY1 |  |  | 400 | 0.907 | 0.3760 | Yes |
| 89 | TGIF1 |  |  | 402 | 0.906 | 0.3789 | Yes |
| 90 | DDR1 |  |  | 403 | 0.906 | 0.3819 | Yes |
| 91 | TTYH1 |  |  | 404 | 0.906 | 0.3850 | Yes |
| 92 | RFTN2 |  |  | 432 | 0.868 | 0.3824 | Yes |
| 93 | PLEKHH2 |  |  | 443 | 0.860 | 0.3833 | Yes |
| 94 | TRIL |  |  | 445 | 0.859 | 0.3860 | Yes |
| 95 | AIF1L |  |  | 456 | 0.851 | 0.3868 | Yes |
| 96 | TRPS1 |  |  | 459 | 0.848 | 0.3893 | Yes |
| 97 | OLFM2 |  |  | 461 | 0.847 | 0.3919 | Yes |
| 98 | LAPTM4B |  |  | 468 | 0.843 | 0.3935 | Yes |
| 99 | IDH1 |  |  | 469 | 0.842 | 0.3964 | Yes |
| 100 | MAML2 |  |  | 470 | 0.841 | 0.3992 | Yes |
| 101 | RNF13 |  |  | 475 | 0.833 | 0.4012 | Yes |
| 102 | ETV1 |  |  | 488 | 0.824 | 0.4015 | Yes |
| 103 | APC |  |  | 492 | 0.822 | 0.4037 | Yes |
| 104 | PSAP |  |  | 501 | 0.816 | 0.4048 | Yes |
| 105 | ARHGEF6 |  |  | 503 | 0.815 | 0.4074 | Yes |
| 106 | DSEL |  |  | 506 | 0.812 | 0.4097 | Yes |
| 107 | ABAT |  |  | 515 | 0.805 | 0.4108 | Yes |
| 108 | PDE4B |  |  | 519 | 0.798 | 0.4129 | Yes |
| 109 | S100A16 |  |  | 521 | 0.797 | 0.4153 | Yes |
| 110 | TNK2 |  |  | 522 | 0.795 | 0.4180 | Yes |
| 111 | DAG1 |  |  | 525 | 0.794 | 0.4203 | Yes |
| 112 | FOS |  |  | 530 | 0.786 | 0.4221 | Yes |
| 113 | ZMYM2 |  |  | 542 | 0.773 | 0.4225 | Yes |
| 114 | NACC2 |  |  | 544 | 0.772 | 0.4249 | Yes |
| 115 | AASS |  |  | 546 | 0.770 | 0.4273 | Yes |
| 116 | SCD5 |  |  | 555 | 0.759 | 0.4282 | Yes |
| 117 | SPATA6 |  |  | 559 | 0.756 | 0.4301 | Yes |
| 118 | WSB1 |  |  | 578 | 0.741 | 0.4290 | Yes |
| 119 | ARHGAP12 |  |  | 581 | 0.739 | 0.4311 | Yes |
| 120 | ILDR2 |  |  | 637 | 0.705 | 0.4223 | Yes |
| 121 | ITGA6 |  |  | 645 | 0.694 | 0.4232 | Yes |
| 122 | TIMP2 |  |  | 650 | 0.691 | 0.4247 | Yes |
| 123 | CSGALNACT1 |  |  | 660 | 0.681 | 0.4252 | Yes |
| 124 | NOTCH1 |  |  | 666 | 0.678 | 0.4264 | Yes |
| 125 | ADCY2 |  |  | 673 | 0.674 | 0.4275 | Yes |
| 126 | FADS2 |  |  | 675 | 0.674 | 0.4296 | Yes |
| 127 | CERS6 |  |  | 690 | 0.664 | 0.4290 | Yes |
| 128 | ITM2C |  |  | 724 | 0.645 | 0.4244 | Yes |
| 129 | MALAT1 |  |  | 739 | 0.638 | 0.4238 | Yes |
| 130 | CREM |  |  | 749 | 0.634 | 0.4241 | Yes |
| 131 | TMEM132B |  |  | 757 | 0.625 | 0.4248 | Yes |
| 132 | PNISR |  |  | 769 | 0.621 | 0.4246 | Yes |
| 133 | GOLIM4 |  |  | 771 | 0.619 | 0.4265 | Yes |
| 134 | CTNND2 |  |  | 775 | 0.618 | 0.4280 | Yes |
| 135 | LRRTM3 |  |  | 782 | 0.613 | 0.4288 | Yes |
| 136 | PBXIP1 |  |  | 792 | 0.607 | 0.4290 | Yes |
| 137 | GAS1 |  |  | 807 | 0.600 | 0.4282 | Yes |
| 138 | JAM2 |  |  | 816 | 0.596 | 0.4286 | Yes |
| 139 | SEMA6D |  |  | 830 | 0.589 | 0.4279 | Yes |
| 140 | SPRED1 |  |  | 837 | 0.585 | 0.4287 | Yes |
| 141 | SAMD8 |  |  | 841 | 0.584 | 0.4300 | Yes |
| 142 | DNASE2 |  |  | 843 | 0.584 | 0.4318 | Yes |
| 143 | TMEM106B |  |  | 846 | 0.582 | 0.4334 | Yes |
| 144 | RHOJ |  |  | 855 | 0.578 | 0.4337 | Yes |
| 145 | SAT1 |  |  | 859 | 0.575 | 0.4350 | Yes |
| 146 | PTPRA |  |  | 862 | 0.575 | 0.4365 | Yes |
| 147 | RAB31 |  |  | 864 | 0.574 | 0.4383 | Yes |
| 148 | SPRY2 |  |  | 871 | 0.573 | 0.4390 | Yes |
| 149 | MLLT6 |  |  | 887 | 0.567 | 0.4378 | Yes |
| 150 | SLITRK2 |  |  | 889 | 0.565 | 0.4395 | Yes |
| 151 | FAM84B |  |  | 892 | 0.564 | 0.4410 | Yes |
| 152 | HIPK2 |  |  | 896 | 0.563 | 0.4423 | Yes |
| 153 | PARP9 |  |  | 897 | 0.562 | 0.4442 | Yes |
| 154 | FAM3C |  |  | 904 | 0.559 | 0.4449 | Yes |
| 155 | ZIC1 |  |  | 911 | 0.556 | 0.4455 | Yes |
| 156 | MT3 |  |  | 913 | 0.555 | 0.4472 | Yes |
| 157 | SLC6A1 |  |  | 927 | 0.549 | 0.4464 | Yes |
| 158 | METTL7A |  |  | 932 | 0.549 | 0.4474 | Yes |
| 159 | BBX |  |  | 939 | 0.547 | 0.4481 | Yes |
| 160 | PCDH17 |  |  | 956 | 0.541 | 0.4467 | Yes |
| 161 | ZMAT3 |  |  | 966 | 0.536 | 0.4466 | Yes |
| 162 | PHLPP1 |  |  | 977 | 0.533 | 0.4464 | Yes |
| 163 | CAMK2D |  |  | 996 | 0.523 | 0.4445 | Yes |
| 164 | SRGAP2B |  |  | 1005 | 0.520 | 0.4446 | Yes |
| 165 | VEGFA |  |  | 1010 | 0.518 | 0.4456 | Yes |
| 166 | ZMAT1 |  |  | 1026 | 0.513 | 0.4443 | Yes |
| 167 | TTC14 |  |  | 1040 | 0.508 | 0.4433 | Yes |
| 168 | RORA |  |  | 1060 | 0.503 | 0.4412 | Yes |
| 169 | NPAS3 |  |  | 1068 | 0.502 | 0.4415 | Yes |
| 170 | C3orf70 |  |  | 1069 | 0.502 | 0.4431 | Yes |
| 171 | ATP1B2 |  |  | 1071 | 0.502 | 0.4446 | Yes |
| 172 | BMPR1A |  |  | 1074 | 0.501 | 0.4459 | Yes |
| 173 | QKI |  |  | 1078 | 0.500 | 0.4470 | Yes |
| 174 | MFGE8 |  |  | 1087 | 0.496 | 0.4470 | Yes |
| 175 | TSPAN3 |  |  | 1093 | 0.494 | 0.4477 | Yes |
| 176 | CD81 |  |  | 1095 | 0.493 | 0.4491 | Yes |
| 177 | IVD |  |  | 1102 | 0.491 | 0.4496 | Yes |
| 178 | PRNP |  |  | 1110 | 0.488 | 0.4498 | Yes |
| 179 | OLIG2 |  |  | 1129 | 0.486 | 0.4478 | No |
| 180 | NRBP2 |  |  | 1133 | 0.485 | 0.4488 | No |
| 181 | GATM |  |  | 1148 | 0.481 | 0.4476 | No |
| 182 | AGPAT3 |  |  | 1154 | 0.479 | 0.4482 | No |
| 183 | NDRG2 |  |  | 1166 | 0.475 | 0.4476 | No |
| 184 | TSPAN7 |  |  | 1181 | 0.471 | 0.4463 | No |
| 185 | VEGFB |  |  | 1183 | 0.470 | 0.4477 | No |
| 186 | NDUFB5 |  |  | 1190 | 0.469 | 0.4480 | No |
| 187 | ZNF621 |  |  | 1200 | 0.465 | 0.4478 | No |
| 188 | CLDND1 |  |  | 1224 | 0.458 | 0.4447 | No |
| 189 | CHP1 |  |  | 1246 | 0.452 | 0.4419 | No |
| 190 | ACOX1 |  |  | 1252 | 0.452 | 0.4424 | No |
| 191 | SNX3 |  |  | 1264 | 0.450 | 0.4417 | No |
| 192 | NDRG4 |  |  | 1266 | 0.449 | 0.4430 | No |
| 193 | PHYHIPL |  |  | 1270 | 0.447 | 0.4439 | No |
| 194 | HS2ST1 |  |  | 1273 | 0.446 | 0.4450 | No |
| 195 | AQP4 |  |  | 1274 | 0.445 | 0.4465 | No |
| 196 | PAX6 |  |  | 1275 | 0.445 | 0.4480 | No |
| 197 | ALDOC |  |  | 1284 | 0.443 | 0.4479 | No |
| 198 | STAT3 |  |  | 1290 | 0.441 | 0.4484 | No |
| 199 | CRYL1 |  |  | 1298 | 0.441 | 0.4484 | No |
| 200 | HEPACAM |  |  | 1319 | 0.436 | 0.4458 | No |
| 201 | LRP4 |  |  | 1331 | 0.433 | 0.4451 | No |
| 202 | BMP7 |  |  | 1368 | 0.423 | 0.4392 | No |
| 203 | ZDHHC21 |  |  | 1371 | 0.423 | 0.4402 | No |
| 204 | LRP1B |  |  | 1395 | 0.417 | 0.4370 | No |
| 205 | SIRPA |  |  | 1408 | 0.415 | 0.4359 | No |
| 206 | HTRA1 |  |  | 1445 | 0.404 | 0.4300 | No |
| 207 | ATP9B |  |  | 1460 | 0.400 | 0.4285 | No |
| 208 | LAMB2 |  |  | 1465 | 0.399 | 0.4290 | No |
| 209 | FYN |  |  | 1483 | 0.394 | 0.4269 | No |
| 210 | GRIA4 |  |  | 1545 | 0.379 | 0.4158 | No |
| 211 | EDNRB |  |  | 1549 | 0.378 | 0.4165 | No |
| 212 | ITPK1 |  |  | 1579 | 0.371 | 0.4119 | No |
| 213 | GLUL |  |  | 1582 | 0.370 | 0.4127 | No |
| 214 | HAPLN1 |  |  | 1612 | 0.366 | 0.4081 | No |
| 215 | SEMA6A |  |  | 1633 | 0.362 | 0.4052 | No |
| 216 | LRRC17 |  |  | 1653 | 0.358 | 0.4026 | No |
| 217 | TOMM7 |  |  | 1686 | 0.353 | 0.3973 | No |
| 218 | RFX4 |  |  | 1707 | 0.348 | 0.3944 | No |
| 219 | CTNNB1 |  |  | 1728 | 0.344 | 0.3915 | No |
| 220 | LRRC4C |  |  | 1737 | 0.342 | 0.3910 | No |
| 221 | TMEM9B |  |  | 1771 | 0.336 | 0.3855 | No |
| 222 | SLC35A5 |  |  | 1776 | 0.335 | 0.3858 | No |
| 223 | NLGN3 |  |  | 1785 | 0.333 | 0.3853 | No |
| 224 | GCSH |  |  | 1791 | 0.332 | 0.3854 | No |
| 225 | SRPK2 |  |  | 1821 | 0.328 | 0.3806 | No |
| 226 | COG2 |  |  | 1856 | 0.322 | 0.3748 | No |
| 227 | NEK6 |  |  | 1912 | 0.312 | 0.3647 | No |
| 228 | TMED10 |  |  | 1946 | 0.306 | 0.3591 | No |
| 229 | MED13 |  |  | 1965 | 0.304 | 0.3565 | No |
| 230 | IRF2 |  |  | 1971 | 0.303 | 0.3565 | No |
| 231 | HMGCS1 |  |  | 1974 | 0.302 | 0.3571 | No |
| 232 | ABCA3 |  |  | 1982 | 0.300 | 0.3567 | No |
| 233 | SEPT2 |  |  | 2017 | 0.294 | 0.3508 | No |
| 234 | SALL2 |  |  | 2026 | 0.292 | 0.3501 | No |
| 235 | ARHGEF26 |  |  | 2029 | 0.292 | 0.3507 | No |
| 236 | PLEKHG1 |  |  | 2050 | 0.290 | 0.3476 | No |
| 237 | SEZ6L |  |  | 2065 | 0.287 | 0.3458 | No |
| 238 | GLDC |  |  | 2077 | 0.286 | 0.3445 | No |
| 239 | SEPT8 |  |  | 2115 | 0.282 | 0.3379 | No |
| 240 | ENHO |  |  | 2173 | 0.272 | 0.3273 | No |
| 241 | PON2 |  |  | 2177 | 0.271 | 0.3276 | No |
| 242 | SLC4A4 |  |  | 2227 | 0.265 | 0.3186 | No |
| 243 | GPC6 |  |  | 2268 | 0.260 | 0.3113 | No |
| 244 | SLC16A1 |  |  | 2282 | 0.258 | 0.3096 | No |
| 245 | ACSS1 |  |  | 2295 | 0.256 | 0.3080 | No |
| 246 | PRKD1 |  |  | 2319 | 0.251 | 0.3042 | No |
| 247 | MCFD2 |  |  | 2343 | -0.251 | 0.3004 | No |
| 248 | B2M |  |  | 2349 | -0.252 | 0.3002 | No |
| 249 | NBPF10 |  |  | 2353 | -0.253 | 0.3005 | No |
| 250 | SMC5 |  |  | 2367 | -0.255 | 0.2987 | No |
| 251 | SCP2 |  |  | 2368 | -0.255 | 0.2995 | No |
| 252 | PPIB |  |  | 2412 | -0.259 | 0.2917 | No |
| 253 | TRAPPC3 |  |  | 2455 | -0.264 | 0.2841 | No |
| 254 | PTPRD |  |  | 2553 | -0.275 | 0.2654 | No |
| 255 | UFL1 |  |  | 2573 | -0.279 | 0.2624 | No |
| 256 | SWAP70 |  |  | 2578 | -0.279 | 0.2626 | No |
| 257 | SEPT7 |  |  | 2703 | -0.295 | 0.2384 | No |
| 258 | JUN |  |  | 2785 | -0.304 | 0.2231 | No |
| 259 | TXNIP |  |  | 2887 | -0.317 | 0.2037 | No |
| 260 | LARS |  |  | 2933 | -0.323 | 0.1956 | No |
| 261 | POLR2L |  |  | 2937 | -0.323 | 0.1961 | No |
| 262 | RIN2 |  |  | 3002 | -0.332 | 0.1843 | No |
| 263 | PRTFDC1 |  |  | 3081 | -0.342 | 0.1696 | No |
| 264 | TJP1 |  |  | 3113 | -0.348 | 0.1645 | No |
| 265 | LIFR |  |  | 3195 | -0.359 | 0.1493 | No |
| 266 | MRPS5 |  |  | 3200 | -0.360 | 0.1497 | No |
| 267 | LINC01138 |  |  | 3208 | -0.361 | 0.1495 | No |
| 268 | S100A13 |  |  | 3219 | -0.363 | 0.1487 | No |
| 269 | PHF11 |  |  | 3224 | -0.364 | 0.1491 | No |
| 270 | PNPT1 |  |  | 3254 | -0.369 | 0.1445 | No |
| 271 | THG1L |  |  | 3312 | -0.377 | 0.1342 | No |
| 272 | FAM120A |  |  | 3335 | -0.380 | 0.1310 | No |
| 273 | TRIM47 |  |  | 3369 | -0.386 | 0.1256 | No |
| 274 | HSPA1A |  |  | 3371 | -0.386 | 0.1267 | No |
| 275 | IGDCC4 |  |  | 3373 | -0.387 | 0.1278 | No |
| 276 | FGF1 |  |  | 3408 | -0.392 | 0.1222 | No |
| 277 | MAST4 |  |  | 3440 | -0.396 | 0.1173 | No |
| 278 | PLOD2 |  |  | 3453 | -0.400 | 0.1162 | No |
| 279 | GNG5 |  |  | 3481 | -0.405 | 0.1121 | No |
| 280 | CCDC80 |  |  | 3492 | -0.408 | 0.1115 | No |
| 281 | TCF7L2 |  |  | 3497 | -0.409 | 0.1120 | No |
| 282 | SDC3 |  |  | 3547 | -0.419 | 0.1035 | No |
| 283 | DIO2 |  |  | 3565 | -0.421 | 0.1015 | No |
| 284 | PCDH7 |  |  | 3576 | -0.423 | 0.1009 | No |
| 285 | CPT1A |  |  | 3665 | -0.437 | 0.0845 | No |
| 286 | LGALS1 |  |  | 3692 | -0.443 | 0.0807 | No |
| 287 | OAT |  |  | 3714 | -0.448 | 0.0780 | No |
| 288 | NTNG1 |  |  | 3719 | -0.449 | 0.0787 | No |
| 289 | CD99 |  |  | 3752 | -0.456 | 0.0737 | No |
| 290 | NBPF14 |  |  | 3796 | -0.465 | 0.0666 | No |
| 291 | PTPN13 |  |  | 3838 | -0.474 | 0.0599 | No |
| 292 | NOTCH2 |  |  | 3853 | -0.477 | 0.0587 | No |
| 293 | APOL2 |  |  | 3925 | -0.493 | 0.0459 | No |
| 294 | RAB13 |  |  | 3933 | -0.494 | 0.0462 | No |
| 295 | ID1 |  |  | 3973 | -0.504 | 0.0400 | No |
| 296 | WDR41 |  |  | 4039 | -0.522 | 0.0285 | No |
| 297 | PSME2 |  |  | 4105 | -0.542 | 0.0172 | No |
| 298 | TACC1 |  |  | 4128 | -0.546 | 0.0146 | No |
| 299 | RHOC |  |  | 4149 | -0.555 | 0.0124 | No |
| 300 | FERMT2 |  |  | 4151 | -0.555 | 0.0141 | No |
| 301 | JAG1 |  |  | 4168 | -0.559 | 0.0127 | No |
| 302 | RGS20 |  |  | 4200 | -0.568 | 0.0083 | No |
| 303 | RCN1 |  |  | 4221 | -0.573 | 0.0062 | No |
| 304 | VIM |  |  | 4255 | -0.582 | 0.0015 | No |
| 305 | IGFBP5 |  |  | 4280 | -0.592 | -0.0014 | No |
| 306 | SLC25A5 |  |  | 4296 | -0.597 | -0.0024 | No |
| 307 | ITGAV |  |  | 4312 | -0.601 | -0.0034 | No |
| 308 | SCCPDH |  |  | 4318 | -0.602 | -0.0024 | No |
| 309 | EFHD2 |  |  | 4390 | -0.632 | -0.0147 | No |
| 310 | OGFRL1 |  |  | 4408 | -0.638 | -0.0160 | No |
| 311 | DGKH |  |  | 4480 | -0.669 | -0.0281 | No |
| 312 | FMNL2 |  |  | 4489 | -0.673 | -0.0275 | No |
| 313 | CYR61 |  |  | 4498 | -0.678 | -0.0268 | No |
| 314 | DAD1 |  |  | 4567 | -0.715 | -0.0382 | No |
| 315 | CST3 |  |  | 4577 | -0.723 | -0.0376 | No |
| 316 | UGP2 |  |  | 4595 | -0.732 | -0.0386 | No |
| 317 | PDLIM5 |  |  | 4612 | -0.739 | -0.0393 | No |
| 318 | LTBP1 |  |  | 4661 | -0.769 | -0.0464 | No |
| 319 | SSR3 |  |  | 4662 | -0.770 | -0.0439 | No |
| 320 | PDXK |  |  | 4708 | -0.801 | -0.0503 | No |
| 321 | ANXA5 |  |  | 4724 | -0.810 | -0.0506 | No |
| 322 | PHACTR2 |  |  | 4733 | -0.817 | -0.0495 | No |
| 323 | APCDD1 |  |  | 4762 | -0.837 | -0.0523 | No |
| 324 | PRDX1 |  |  | 4783 | -0.848 | -0.0535 | No |
| 325 | GLUD1 |  |  | 4786 | -0.849 | -0.0511 | No |
| 326 | SERPINH1 |  |  | 4829 | -0.895 | -0.0566 | No |
| 327 | SPATS2L |  |  | 4833 | -0.901 | -0.0541 | No |
| 328 | ZFP36L2 |  |  | 4863 | -0.926 | -0.0569 | No |
| 329 | PSAT1 |  |  | 4867 | -0.932 | -0.0544 | No |
| 330 | SLC2A1 |  |  | 4949 | -1.018 | -0.0674 | No |
| 331 | ZFP36L1 |  |  | 4969 | -1.040 | -0.0677 | No |
| 332 | EPB41L2 |  |  | 4974 | -1.051 | -0.0650 | No |
| 333 | PKM |  |  | 4986 | -1.071 | -0.0636 | No |
| 334 | NOTCH3 |  |  | 5003 | -1.098 | -0.0632 | No |
| 335 | ADAMTS6 |  |  | 5017 | -1.121 | -0.0620 | No |
| 336 | NTM |  |  | 5052 | -1.188 | -0.0649 | No |
| 337 | HOPX |  |  | 5056 | -1.204 | -0.0615 | No |
| 338 | DLC1 |  |  | 5057 | -1.204 | -0.0574 | No |
| 339 | MT2A |  |  | 5061 | -1.216 | -0.0539 | No |
| 340 | TIMP1 |  |  | 5086 | -1.257 | -0.0546 | No |
| 341 | ALCAM |  |  | 5118 | -1.327 | -0.0564 | No |
| 342 | MT1E |  |  | 5129 | -1.359 | -0.0538 | No |
| 343 | ENO1 |  |  | 5146 | -1.410 | -0.0523 | No |
| 344 | LRP1 |  |  | 5152 | -1.437 | -0.0485 | No |
| 345 | AXL |  |  | 5165 | -1.518 | -0.0458 | No |
| 346 | COL11A1 |  |  | 5169 | -1.526 | -0.0413 | No |
| 347 | PHGDH |  |  | 5170 | -1.528 | -0.0362 | No |
| 348 | TFPI |  |  | 5212 | -1.705 | -0.0388 | No |
| 349 | F3 |  |  | 5230 | -1.856 | -0.0360 | No |
| 350 | EGFR |  |  | 5233 | -1.904 | -0.0300 | No |
| 351 | SLC7A11 |  |  | 5257 | -2.165 | -0.0273 | No |
| 352 | PTCH1 |  |  | 5261 | -2.318 | -0.0201 | No |
| 353 | RGCC |  |  | 5264 | -2.349 | -0.0126 | No |
| 354 | SSPN |  |  | 5273 | -2.514 | -0.0058 | No |
| 355 | COL6A1 |  |  | 5278 | -2.689 | 0.0024 | No |
Table: GSEA details [plain text format]

  

Fig 2: RICHARDS\_NOWAKOWSKI\_SCIENCE\_2017\_ASTROCYTE\_UPREG: Random ES distribution      
 Gene set null distribution of ES for **RICHARDS\_NOWAKOWSKI\_SCIENCE\_2017\_ASTROCYTE\_UPREG**
